# Risk Factors for Development of Hemolytic Uremic Syndrome in a Cohort of Adult Patients with STEC 0104:H4 Infection

**DOI:** 10.1371/journal.pone.0059209

**Published:** 2013-03-22

**Authors:** Alexander Zoufaly, Jakob P. Cramer, Eik Vettorazzi, Friedhelm Sayk, Jan P. Bremer, Irmtraut Koop, Andreas de Weerth, Stefan Schmiedel, Sabine Jordan, Katharina Fraedrich, Niels H. Asselborn, Martin Nitschke, Christine Neumann-Grutzeck, Tim Magnus, Christoph Rüther, Klaus Fellermann, Rolf K. Stahl, Karl Wegscheider, Ansgar W. Lohse

**Affiliations:** 1 Department of Medicine I (Gastroenterology and Hepatology, Infectious Diseases and Tropical Medicine), University Medical Center Hamburg-Eppendorf, Hamburg, Germany; 2 Department of Medical Biometry and Epidemiology, University Medical Center Hamburg-Eppendorf, Hamburg, Germany; 3 Department of Internal Medicine I , University of Schleswig-Holstein, Campus Lübeck, Lübeck, Germany; 4 I. Medical Department, Asklepios Klinik Altona, Hamburg, Germany; 5 Department of Medicine, Amalie Sieveking Hospital, Hamburg, Germany; 6 Department of Internal Medicine, Agaplesion Diakonieklinikum, Hamburg, Germany; 7 Department of Neurology, University Medical Center Hamburg-Eppendorf, Hamburg, Germany; 8 Department of Medicine III, University Medical Center Hamburg-Eppendorf, Hamburg, Germany; University of Birmingham, United Kingdom

## Abstract

The outbreak of Shiga toxin producing E.coli O104:H4 in northern Germany in 2011 was one of the largest worldwide and involved mainly adults. Post-diarrheal hemolytic uremic syndrome (HUS) occurred in 22% of STEC positive patients. This study’s aim was to assess risk factors for HUS in STEC-infected patients and to develop a score from routine hospital parameters to estimate patient risks for developing HUS. In a cohort analysis, adult patients with STEC infection were included in five participating hospitals in northern Germany between May and July 2011. Clinical data were obtained from questionnaires and medical records, laboratory data were extracted from hospitals’ electronic data systems. HUS was defined as thrombocytopenia, hemolytic anemia and acute renal dysfunction. Random forests and multivariate logistic regression were used to identify risk factors for HUS and develop a score using the estimated coefficients as weights. Among 259 adults with STEC infection, vomiting (OR 3.48,95%CI 1.88–6.53), visible blood in stools (OR 3.91,95%CI1.20–16.01), age above 75 years (OR 3.27, 95%CI 1.12–9.70) and elevated leukocyte counts (OR 1.20, 95%CI 1.10–1.31, per 1000 cells/mm^3^) were identified as independent risk factors for HUS. A score using these variables has an area under the ROC curve of 0.74 (95%CI 0.68–0.80). Vomiting, visible blood in stools, higher leukocyte counts, and higher age indicate increased risk for developing HUS. A score using these variables might help to identify high risk patients who potentially benefit from aggressive pre-emptive treatment to prevent or mitigate the devastating consequences of HUS.

## Introduction

The hemolytic uremic syndrome (HUS) is a serious and potentially life-threatening complication in infections with Shiga toxin-producing Escherichia coli (STEC) [1], [2]. While most cases of uncomplicated STEC-associated hemorrhagic colitis recover after a few days without sequelae, 3–22% proceed to HUS which may be further complicated by neurological symptoms and death [2], [3]. In retrospective studies of outbreaks of *E. coli* O157:H7 infection, age and elevated white blood cell count (WBC) have been consistently reported as risk factors for HUS although most data are available for children [4], [5], [6], [7], [8]. Data on clinical predictors for HUS concerning adults are scarce and mostly derived from sporadic outbreaks involving O157 and O111 serotypes [4], [9].

The 2011 outbreak in northern Germany was caused by the Shiga toxin 2 (variant vtx 2a) producing serovar O104:H4 [10] and differed substantially from previous outbreaks caused by other STEC strains regarding the high proportion (88%) of adults and the high virulence resulting in an unprecedented number of HUS cases [11]. During the outbreak with this novel strain, patients with symptoms of uncomplicated colitis and proven STEC infection were followed daily mostly as inpatients at secondary and tertiary health care centers until complete recovery or the onset of HUS and other complications. This close follow-up was mandatory due to the lack of established prognostic markers and risk factors. However, this task is associated with massive impact on both, patients and health care workers and causes high costs [12]. As currently no causal therapy for established HUS exists, early identification of patients with STEC infection at high risk for complications is essential for the provision of pre-emptive treatment to prevent development of HUS and to facilitate the clinical management.

In the present study, we analyzed data from a multi-center cohort of adult patients with confirmed O104:H4 STEC-infection who were followed-up until complete recovery or onset of HUS. We aimed to identify risk factors for HUS and to develop a score which could serve as a risk stratification tool in the management of future cases.

## Methods

### Ethics Statement

Approval for the performance of the study was granted by the *Ethics Committee* of the *Hamburg* and Lübeck Chamber of Physicians. Written consent was obtained from study participants.

### Selection of the Study Population

Patients 18 years of age and older with microbiologically confirmed STEC infection and with at least one clinical contact at one of the participating centers between May 1^st^ and July 1^st^ 2011 were included in this cohort study after written informed consent was obtained. Participating centers included the University Medical Center Hamburg-Eppendorf, the Asklepios Klinik Altona, the Amalie Sieveking hospital, and the Agaplesion Diakonieklinikum in Hamburg, and the University Medical Center in Lübeck, northern Germany. In the participating centers patients were diagnosed and treated in emergency departments which had set up specialized isolation and treatment units. The participating hospitals provided care for the majority of patient with STEC infection within the cities of Hamburg and Lübeck which reported the highest incidences in this outbreak [11]. The availability of these hospital based treatment units was communicated to general practitioners as well as to the public by local media. Therefore patients seen at the treatment units of participating centers included self-referrals as well as referrals by GPs.

Data regarding clinical symptoms and possible risk factors for STEC infection were collected at first presentation for medical care in the participating centers by physicians involved in the research using questionnaires. In patients who could not be initially assessed because of rapid deterioration, missing information was collected in retrospect from medical records. Questionnaires for the assessment of symptoms were developed in each center at the onset of the outbreak. These questionnaires were later standardized across centers and a common database was established. Patients were invited for a clinical follow-up visit or telephone consultation at least two weeks after first presentation at a participating center. At that point, patients were asked to provide information about the total duration of symptoms such as bloody or non-bloody diarrhea, the occurrence of complications, as well as additional information about risk behavior which had not been assessed initially. Laboratory data were recorded at initial presentation and at all follow-up visits. Completion and verification of clinical data was performed from hospital electronic data systems, medical files, and HUS registers.

### Definition of STEC Infection and HUS

Patients were considered to be infected with the outbreak STEC-strain if stool cultures were tested positive for ESBL-producing E. coli that were positive for Stx 2 and negative for Stx 1 using PCR or ELISA during the outbreak period. HUS was defined as the combination of thrombocytopenia (platelets below 150.000 billion/L) and hemolytic anemia (hemoglobin below the lower limit of normal and lactate dehydrogenase levels above normal or presence of blood fragmentocytes on a peripheral blood smear) and evidence of acute renal failure (rise of the creatinine level above age- and sex-adjusted normal levels, oliguria, anuria, proteinuria, or hematuria) [13], [14].

Patients who had evidence for HUS at first presentation were excluded from analyses. Further exclusion criteria were missing laboratory test within two days of first presentation as well as an initial hospital presentation outside the participating centers with subsequent referral for specialized treatment. If multiple laboratory tests were available the test closest to the date of first presentation was selected for inclusion in the analysis.

### Statistical Analyses

Characteristics of patients with proven STEC infection were compared using Student’s t-test for normally distributed data and Mann Whitney-U test otherwise. Categorical data were compared using the chi-squared test.

Random forests were used to identify risk factors for the development of HUS among a predefined set of variables which were thought to be potentially associated with development of HUS. The choice of variables was made based on previous reports, our own exploratory analyses, as well as biologic plausibility. Variables found to be important in the random forest analysis were used to construct a decision tree for the classification of patients with and without HUS [15]. Missing laboratory values were imputed if at least 80% of values were available in the dataset using the methods provided within the random forest package [16], [17]. Variables selected for analyses included age (in two categories), sex, vomiting, presence of bloody stools, abdominal pain, stool frequency, underlying comorbidities, C-reactive protein, leukocytes, and hemoglobin levels.

In a second step, variables found to be associated with HUS in the random forest analysis were re-assessed in multivariate logistic regression models in an attempt to gain a highly sensitive model. The final score was developed using estimated coefficients of the last model as weights. A receiver operating characteristics curve was created to illustrate sensitivity and specificity of the score.

In a sensitivity analysis we restricted our model to patients who presented for care at a maximum of 4 days after developing the first episode of diarrhea. Patients without diarrhea were excluded in this analysis. A value of p<0.05 was specified for significance. All analyses were done using R version 2.13.2 [18].

## Results

From May ^18th^ to July 1^st^ 2011, a total of 443 patients were tested positive for Stx2-/ESBL-producing STEC in the five participating centers. Informed consent was provided by 315 patients, 67 patients were excluded for missing laboratory tests or presence of HUS at first contact or the fact that they were referred with known STEC infection from other hospitals for specific treatment of suspected HUS. A total of 259 patients with confirmed STEC infection and without evidence of HUS at first presentation were included. Mean age was 44.4 (standard deviation, SD, = ±17.1) years and 64.1% of patients were female. While 70.7% of patients were admitted to medical wards, the remaining patients received follow-up care in outpatient departments or by their general practitioners.

At first presentation, 255 (98.5%) patients reported diarrhea and 4 (1.5%) patients reported close contact with other STEC cases and had abdominal pain or loose stools without fulfilling the criteria for diarrhea (≥3 stools loose stools per day). Median duration of diarrhea was 6 (IQR 4–9) days and was longer in patients developing HUS (median 6 vs. 7 days, p = 0.051). Further frequent presenting symptoms were bloody stools in 234 (90.3%) and vomiting in 71 (27.6%) patients. Abdominal pain was reported in 234 (91.1%) patients without a clear preponderance in those with and without HUS. Only Concomitant chronic diseases were present in 96 (37.2%) individuals with arterial hypertension (17.4%), hypothyroidism (5.1%), and chronic renal disease (3.1%) being the most common. Median time from first episode of diarrhea to first presentation in a hospital was 2 (IQR 1–4) days and 81% of patients presented within 4 days. This time was shorter in patients who developed HUS (median 1 day, IQR 1–3 days) compared to patients who did not develop HUS (median 2 days, IQR 1–4, p = 0.016). Characteristics of patients with proven STEC infection and laboratory tests at first presentation to a health care center are summarized in table 1 and table 2. Laboratory tests were available on the day of first presentation in 86% of patients, within one day of first presentation in 11% and within 2 days in 3%. A total of 81 (31.3%) patients developed HUS. Median time to HUS after first episode of diarrhea was 5 (IQR 3–6) days. Time to HUS was at a median of 4 (IQR 3–6) days after first medical contact, and similarly 4 days (IQR 3–6, p = 0.13) after first available laboratory test. Independent risk factors for the development of HUS in the multivariable model included vomiting (OR 3.48, 95%CI 1.88–6.53, p<0.001), age above 75 years (OR 3.27, 1.12–9.70, p = 0.030), and visible blood in stools (OR 3.91, 95%CI 1.2–16.0, p = 0.036). In addition, higher leukocyte counts were significantly associated with higher risk for development of HUS (OR 1.12 per 1000 cells/mm^3^, 95%CI 1.10–1.31, p<0.001, table 3). A sensitivity analysis including only patients who presented for medical care within 4 days of first episode of diarrhea yielded similar point estimates compared to the analysis including all patients (OR for vomiting 3.46, 95%CI 1.87–6.48, p<0.001, OR for leukocytes per 1000 cell/ mm^3^ higher 1.19,95%CI 1.10.–1.31 p<0.001, OR for visible blood in stool 3.15, 95%CI 0.92–13.23, p = 0.086, and OR for age>75 years 3.23, 95%CI 1.11–9.55, p = 0.028).

**Table 1:** Characteristics of patients treated for STEC infection without evidence for hemolytic uremic syndrome (HUS) at first presentation to health care center.

			Development of Hemolytic Uremic Syndrome (HUS)		
		total	no	yes	
Characteristic		n = 259	n = 178 (68.7%)	n = 81 (31.3%)	p-value*
Hospital – no. (% of total)					0.096
University Medical Center Hamburg-Eppendorf		105 (40.5)	77 (43.3)	28 (34.6)	
Asklepius Klinik Altona, Hamburg		42 (16.2)	23 (12.9)	19 (23.5)	
Agaplesion Diakoniekrankenhaus Hamburg		9 (3.5)	8 (4.5)	1 (1.2)	
Amalie Sieveking Hospital Hamburg		22 (8.5)	17 (9.6)	5 (6.2)	
University Medical Center Lübeck		81 (31.3)	53 (29.8)	28 (34.6)	
Admission to hospital - no. (%)		183 (70.7)	102 (57.3)	81 (100.0)	< 0.001
Age (first presentation), mean years± SD		44.4 ± 17.1	43.7 ± 16.7	45.8 ± 18.0	0.447
Female Sex - no. (%)		166 (64.1)	109 (61.2)	57 (70.4)	0.152
Pregnancy - no./total no. (%)		5/164 (3.0)	3/108 (2.8)	2/56 (3.6)	0.782
Diarrhea - no. (%)		255 (98.5)	174 (97.8)	81 (100.0)	0.082
Visible Blood in stools - no. (%)		234 (90.3)	157 (88.2)	77 (95.1)	0.067
Abdominal pain - no./total no. (%)		234/257 (91.1)	156/176 (88.6)	78/81 (96.3)	0.032
Vomiting - no./total no. (%)		71/257 (27.6)	35/176 (19.9)	36/81 (44.4)	< 0.001
Premorbid conditions - no./total no. (%)		96/258 (37.2)	65/177 (36.7)	31/81 (38.3)	0.811
Intake of Oral contraceptive pill no./total no. (%)		42/142 (29.6)	27/91 (29.7)	15/51 (29.4)	0.974
Use of antibiotics within 2 weeks prior to presentation –no./total no. (%)		5/135 (3.7)	3/101 (3.0)	2/34 (5.9)	0.459
Use of antimotility agents within 2 weeks prior to presentation –no./total no. (%)		11/133 (8.3)	11/102 (10.8)	0/31 (0.0)	0.013

* Student’s t-test and chi-squared test.

**Table 2:** Laboratory values in patients without evidence of hemolytic uremic syndrome (HUS) at first presentation to health care center.

			Development of Hemolytic Uremic Syndrome (HUS)		
		total	no	yes	
Laboratory value at first presentation		n = 259	n = 178 (68.7%)	n = 81 (31.3%)	p-value*
Sodium – mean mmol/L ± SD (% valid)		138.22 ± 3.43 (75.3)	138.68 ± 3.44 (82.0)	136.84 ± 3.02 (60.5)	< 0.001
Potassium - mean mmol/L ± SD (% valid)		3.81 ± 0.37 (75.3)	3.80 ± 0.37 (82.0)	3.82 ± 0.38 (60.5)	0.761
ALAT – mean µkat/L ± SD (No. valid)		0.35 ± 0.19 (78.0)	0.35 ± 0.20 (81.5)	0.32 ± 0.19 (70.4)	0.170
ASAT – mean µkat/L ± SD (% valid)		0.36 ± 0.12 (81.1)	0.36 ± 0.11 (84.8)	0.35 ± 0.13 (72.8)	0.540
Albumine – mean g/L± SD (No. valid)		35.81 ± 4.33 (20.1)	36.57 ± 4.44 (19.1)	34.38 ± 3.83 (22.2)	0.134
Bilirubine, total – mean µmol/L ± SD (% valid)		13.34 ± 8.55 (49.8)	12.66 ± 8.38 (53.4)	14.88 ± 8.72 (42.0)	0.192
Blood urea – mean mmol/L ± SD (% valid)		5.14 ± 3.45 (61)	5.06 ± 2.54 (65.2)	5.35 ± 5.24 (51.9)	0.192
Creatinine mean µmol/L ± SD (% valid)		74.26 ± 20.33 (100)	75.14 ± 18.56 (100)	71.60 ± 23.87 (100)	0.022
Lipase – mean µkat/L ± SD (% valid)		0.39 ± 0.37 (47.5)	0.43 ± 0.44 (41.6)	0.31 ± 0.20 (60.5)	0.032
Lactate dehydrogenase – mean µkat/L ± SD (% valid)		3.00 ± 0.75 (94.2)	2.98 ± 0.76 (96.1)	3.06 ± 0.74 (90.1)	0.439
C-reactive protein – mean nmol/L (SD,% valid)		18.0 (28.7, 98.8)	14.2 (19.7, 98.3)	26.3 (41.1, 100)	0.014
Hemoglobin – mean g/dL ± SD (% valid)		14.14 ± 1.51 (99.6)	14.10 ± 1.51 (99.4)	14.24 ± 1.53 (100)	0.664
Leukocytes mean cells/mm^3^ ± SD (% valid)		10.90 ± 3.63 (99.6)	10.17 ± 3.12 (99.4)	12.49 ± 4.13 (100)	< 0.001
Platelets - mean billion/L ± SD (% valid)		244 ± 53 (99.2)	239 ± 52 (99.4)	254 ± 54 (98.8)	0.062

* Mann-Whitney U test.

**Table 3:** Multivariable logistic regression model analyzing risk factors for hemolytic uremic syndrome and risk score to predict development of HUS in patients with proven STEC infection (add points to get total risk score).

	OR	95% CI		P	Risk scorepoints
Vomiting	3.48	1.88	6.53	<0.001	1.2
Age ≥75 years*	3.27	1.12	9.70	0.030	1.2
Visible blood in stools	3.91	1.20	16.01	0.036	1.4
Leukocytes (per 1000cells/mm^3^ higher)	1.12	1.10	1.31	<0.001	0.2

* vs. <75 years.

**Figure 1:**
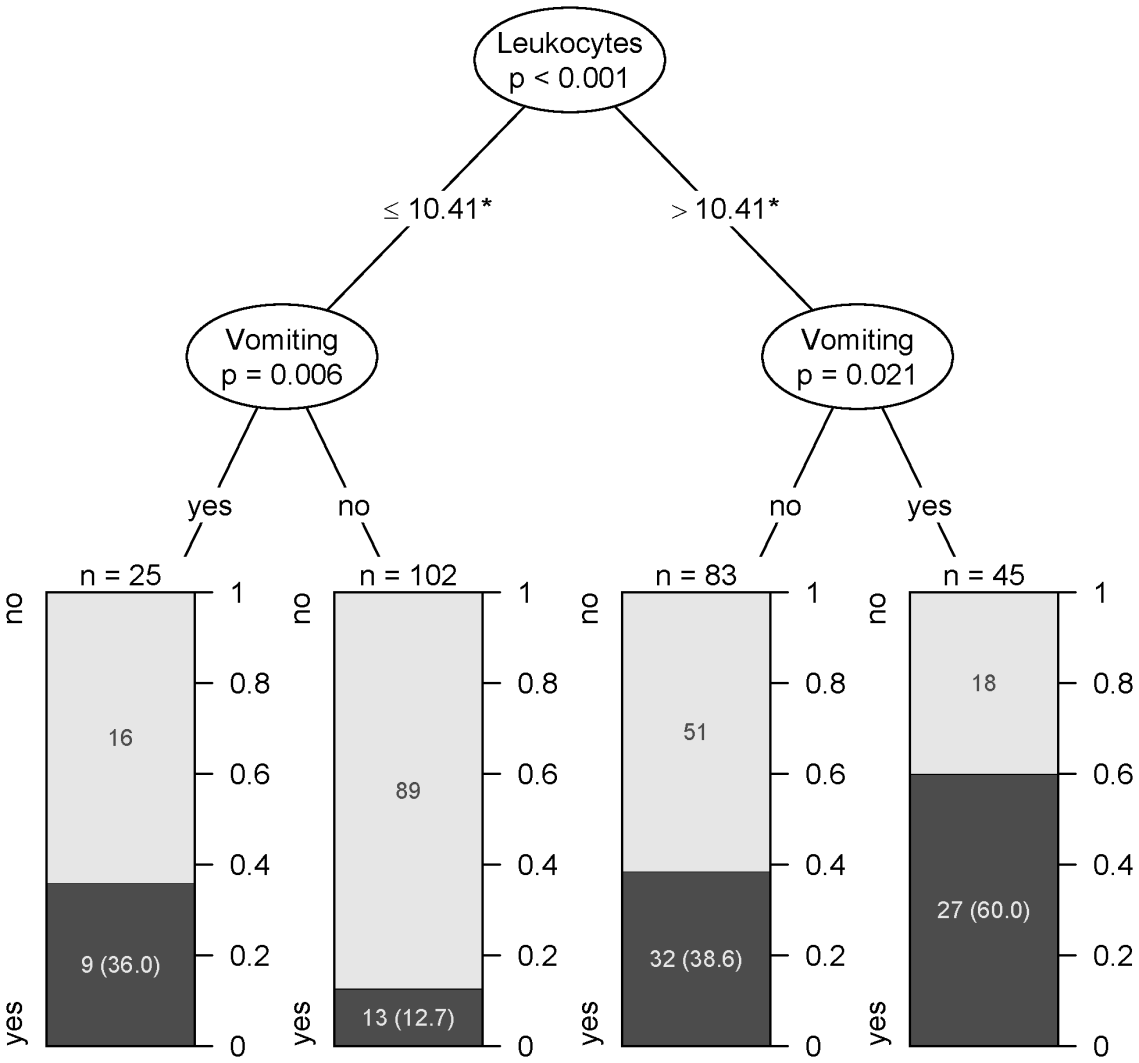
Decision tree allowing classification of patients with HUS (dark) and without HUS (bright). * cells /cubic mm.

Leukocytes above 10.410 cells/mm^3^ and presence of vomiting were the most important variables and had the highest performance to classify patients regarding risk for HUS in a recursive partitioning analysis using random forests (figure 1).

**Figure 2:**
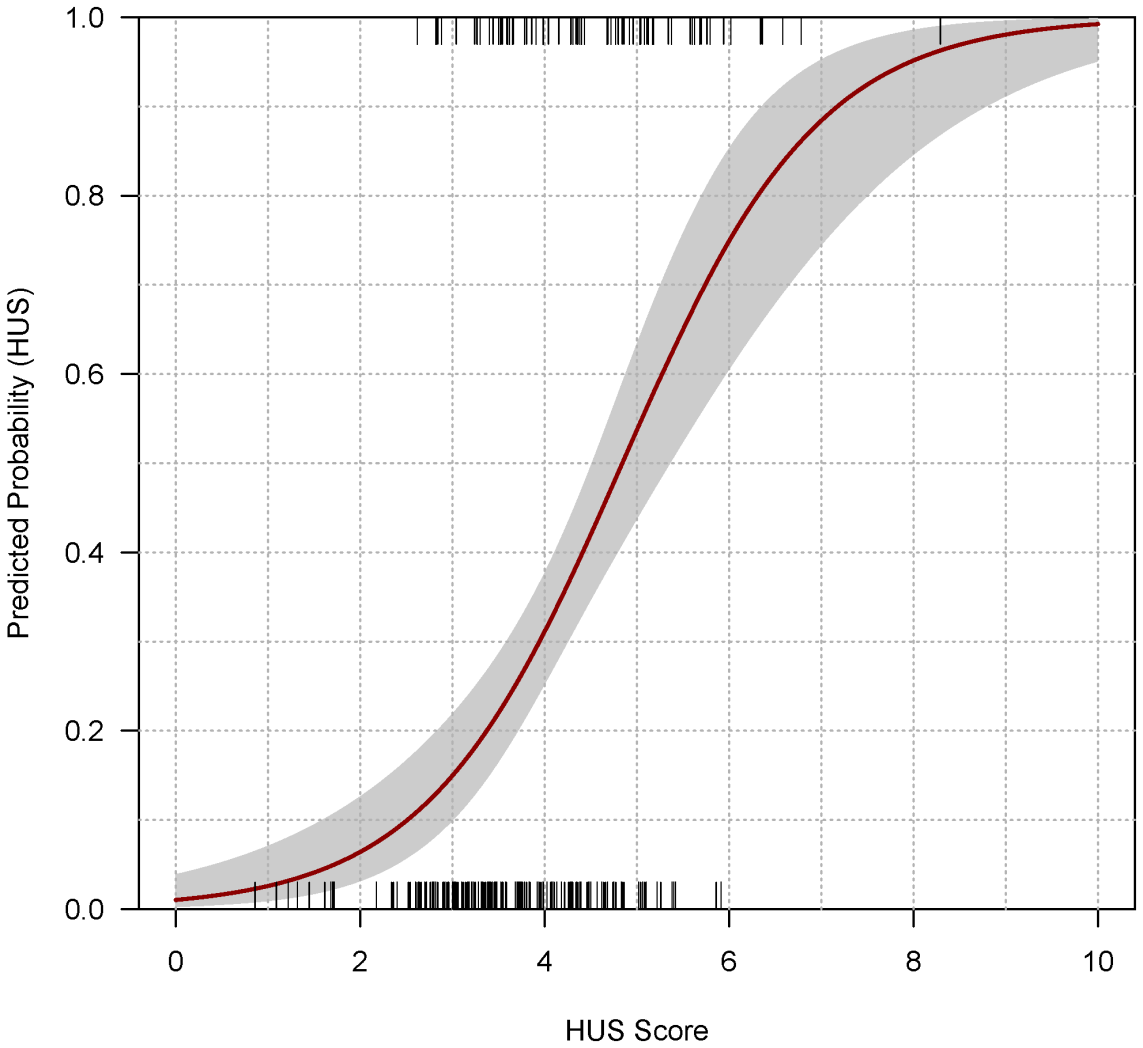
Predicted probability for HUS according to risk score. Grid (“rug”) above indicates observed HUS cases and corresponding scores, grid below indicates patients without HUS and corresponding scores. 95% confidence interval is in grey shades.

**Figure 3:**
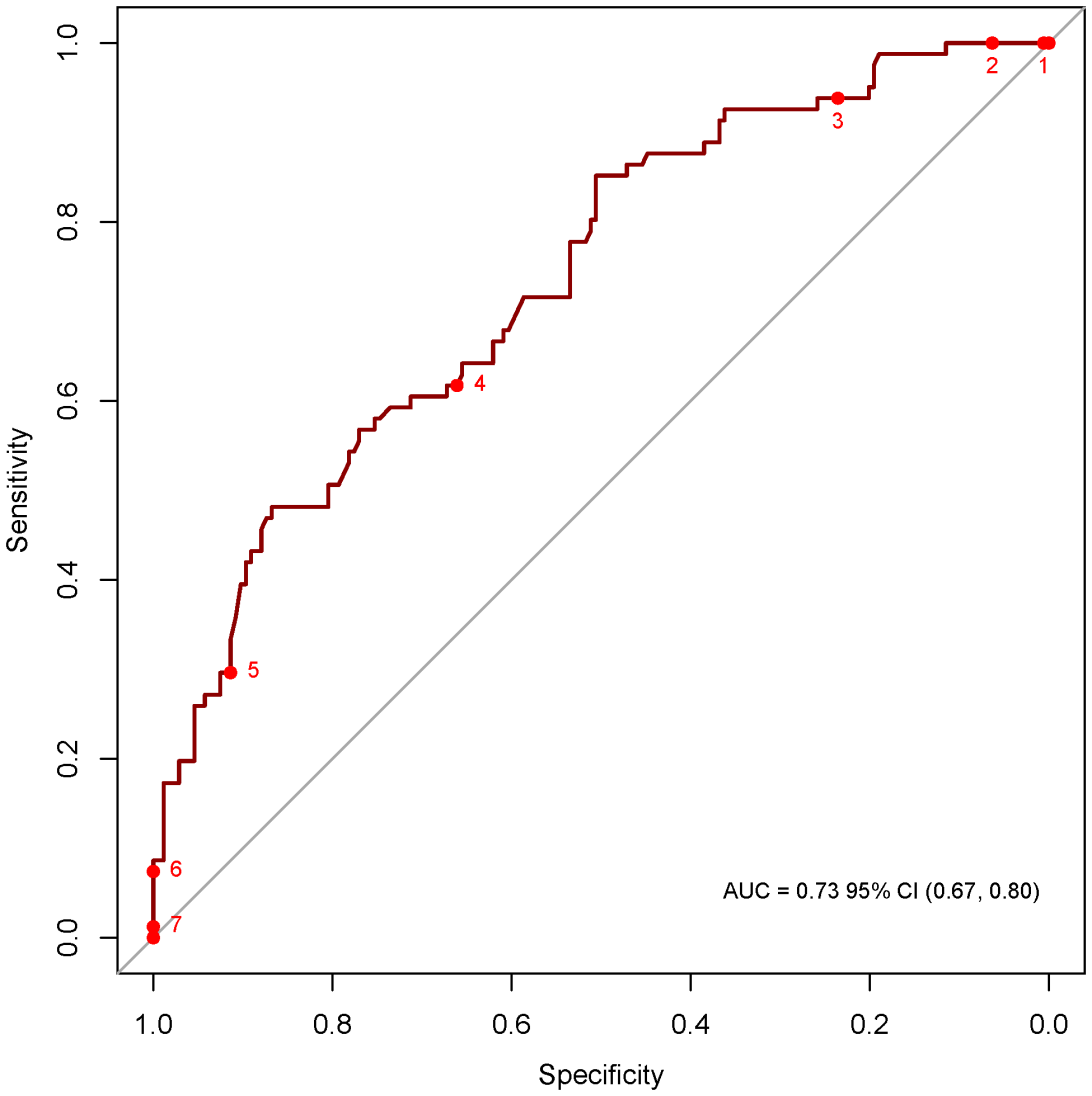
Receiver operating characteristic (ROC) curve indicating specificity and sensitivity of the risk score (Numbers indicate score-cutoffs).

From the results of the multiple logistic regression analysis, a score was developed. This score used the coefficients found in the multiple logistic regression as weights. In accordance with the final logistic regression model, factors used in the score included vomiting (1.2 points since the coefficient for vomiting was 1.24 in the multivariate model), age above 75 years (1.2 points), visible blood in stools (1.4 points), and leukocyte counts (0.2 points per 1000 cells/mm^3^ higher). The score ranges from 0 (very low risk for developing HUS) to a maximum of 10 points (very high risk). Below a score of 2.60 no case of HUS was observed in 23 (8.9%) patients, above a score of 6.0, all 6 (2.3%) patients developed HUS. Figure 2 shows the predicted probability of HUS according to the score and observed cases of HUS. The receiver operating characteristic (ROC) curve of the score was 0.74 (95%CI 0.68–0.80) and is depicted in figure 3. Specificity and sensitivity of the score at various cutoffs are depicted in table 4.

**Table 4:** Specificity and sensitivity of the risk score.

Score threshold	specificity	sensitivity	Positive predictive value	Negative predictivevalue	True positive	True negative	False negative	False positive
0	0.000	1.000	0.313		81	0	0	178
1	0.006	1.000	0.314	1.000	81	1	0	177
2	0.079	1.000	0.331	1.000	81	14	0	164
3	0.247	0.938	0.362	0.898	76	44	5	133
4	0.663	0.617	0.455	0.792	50	118	31	60
5	0.921	0.296	0.632	0.742	24	164	57	14
6	1.000	0.074	1.000	0.704	6	178	75	0
7	1.000	0.012	1.000	0.690	1	178	80	0
8	1.000	0.012	1.000	0.690	1	178	80	0
9	1.000	0.000		0.687	0	178	81	0
10	1.000	0.000		0.687	0	178	81	0

## Discussion

We identified vomiting, elevated leukocyte counts, age >75years, and presence of visible blood in stools as independent risk factors for the development of HUS in adults infected with the novel E.coli STEC O104:H4 strain. Our data are derived from the largest HUS outbreak to date and suggest a strong positive linear correlation between leukocyte counts and HUS. The presence of elevated leukocytes could indicate a host inflammatory response to Stx-production [19]. Both, leukocytes and vomiting have been previously associated with risk for HUS in patients infected with E.coli O157:H7 [4], [7] and O111 [9]. This potentially suggests that host risk factors may not differ entirely by strain. Hence, the score which is based on risk factors found in this O104 outbreak may provide useful information and could potentially be evaluated in future outbreaks involving other STEC strains as well.

Additional risk factors identified in our study included older age with a cutoff at 75 years and presence of bloody diarrhea. Possibly, pre-existing renal damage or subclinical renal insufficiency as well as relative dehydration – conditions frequently present in older age groups – may contribute to the development of HUS. Furthermore, age-related changes of gut flora and, possibly, altered mucosal immune defense mechanisms as well as prolonged intestinal transit may play a role [20]. Impaired intestinal barrier function could facilitate bacterial invasion and Stx-uptake and this could explain the association of bloody diarrhea with increased risk of HUS. Interestingly, the proportion of bloody diarrhea was lower in children who developed HUS in the same outbreak compared to adults. [11], [21] Furthermore, in a previous study, involving O157 infected children with and without HUS, rates of bloody diarrhea were similar. [7] This supports a possible association between intestinal barrier function and Stx-uptake and underlines distinct properties of this novel O104 strain compared to O157 [10].

Early identification of patients at risk for HUS is necessary particularly in outbreak scenarios where a high number of patients with a potentially life-threatening condition seek medical care and capacities for close monitoring of all patients infected with STEC may be lacking. In addition, it may be prudent to prioritize novel treatment strategies for patients at highest risk. In our analysis, time from onset of symptoms to first presentation in the hospital was shorter in patients who later developed HUS, which is probably reflecting the higher severity of symptoms in these patients. As previously described, the time between onset of gastrointestinal symptoms and HUS in this outbreak was only 5 days indicating a short window of opportunity to timely identify high-risk patients and to potentially initiate early preventive treatment [11]. Leukocytes together with presence of vomiting had the best performance to discriminate between patients with and without HUS: Among patients with vomiting and a leukocytes count higher than 10.410 cells/mm^3^ 60% developed HUS. To further increase sensitivity and specificity we tried to develop a risk- score allowing a better discrimination between risk groups.

23 patients in our cohort had a HUS score ≤2.60 and none (95%CI 0–15%) developed HUS. Stringent follow-up including daily blood analyses may not be necessary in these patients. However, given the low number of patients in this subgroup and the large confidence interval it is currently not justified to conclude that these patients are at no risk for HUS.

A HUS score of 3.0–4.0 was the most frequently observed in our patients. This score corresponds to a probability of HUS between 17% and 36% which is in line with the HUS rate of 22% among STEC positive patients reported through the German surveillance system [11].

Out of 38 patients with a score ≥5.0, 24 (63.2%, 95%CI 46.0–78.2%) developed HUS. All patients (95%CI 59–100%) with a score ≥6.0 developed HUS.

Potentially in these high risk patients development of HUS could have been averted by early aggressive measures to reduce intestinal bacterial and toxin load. It is possible that strategies including intravenous volume expansion [22]and, if tolerated, gastrointestinal washing with macrogol may have a prophylactic effect if provided early and reduce stx release before intravascular thrombin generation occurs which heralds the hemolytic uremic syndrome [23]. This may be particularly true given the enteroaggregative properties of this novel strain.

STEC infection and HUS are highly dynamic diseases and all clinical decisions need to relate to the time in illness at which patients present for care. In a sensitivity analysis we therefore restricted our model to patients who presented early (within 4 days) for medical care. However, the estimates yielded were similar to the ones in the main analysis.

Rather than identifying patients at no risk, our score allows classification of patients at particularly high risk for developing HUS who should receive early and aggressive preemptive treatment. Until more specific markers to further stratify patients at low and intermediate risk have become available and this score has been validated in further studies we recommend routine follow-up for all patients with proven STEC infection. Pre-emptive therapy that is not associated with risks or adverse events should not be withheld based upon perception of no risk.

The present study has some limitations: Firstly, a large number of patients had to be excluded due to incomplete laboratory data or missing consent which may inflict selection bias. Moreover, only larger hospitals including two university medical centers contributed data to the study and patients with HUS at first medical contact were excluded in this analysis of prospective risk factors. Milder cases possibly did not report to medical services or were exclusively treated by general practitioners and, hence, may have been underreported. On the other hand, patients could have declined participation due to severe disease which could have led to insufficient capture of severe cases. However, the overall HUS rate in our study is even higher than the national reported rate and the high proportion of outpatient cases (29.3%) in our study adds valuable information about patients with STEC infection and lower risk for HUS. Secondly, the questionnaires initially used were not standardized leaving some of the data incomplete. Some patients who presented to participating hospitals deteriorated quickly before they could be assessed by a physician involved in the research. In those cases, missing data were retrospectively collected as soon as possible. However, information on the risk factors analyzed in our model was consistently available in all individual cohorts. The outbreak was unexpected and enrollment into the study and harmonization of data collection could only start after ethical clearance was obtained. Nevertheless, the demographic structure of our study population is comparable to the nationwide outbreak description. Lastly the proposed risk score is only valid for the population studied and without validation cannot be extrapolated to outbreaks including different strains, sporadic cases, or other populations such as children. Further validation by an independent data set is particularly necessary before the risk stratification score should be used in a wider clinical context.

In conclusion, this analysis identifies vomiting, visible blood in stools, age>75 years and higher leukocyte counts as risk factors for development of HUS among adults infected with STEC O104:H4. A risk score including these four factors may help identify high risk patients who are at risk for imminent HUS and require aggressive preemptive treatment to prevent or mitigate the devastating consequences of HUS.
