# Correction: Risk Factors for Development of Hemolytic Uremic Syndrome in a Cohort of Adult Patients with STEC 0104:H4 Infection

**DOI:** 10.1371/journal.pone.0091617

**Published:** 2014-03-07

**Authors:** The PLOS ONE Staff

The first two authors, Alexander Zoufaly and Jakob P. Cramer, should be noted as having contributed equally to the article.

In addition, a funding organization and grant are incorrectly omitted from the Funding statement. The following sentence should be included with the Funding statement: “This research was funded by a grant from the German Ministry of Health (BMG).”
